# Frequency of dentoalveolar trauma in high-performance athletes: a systematic review in multi-sport events in Latin America

**DOI:** 10.12688/f1000research.169394.1

**Published:** 2026-04-18

**Authors:** Felipe Avendaño, Catalina Opazo-García, Jeel Moya-Salazar, Juan Pablo Morales, Bárbara Salgado, Hans Contreras-Pulache

**Affiliations:** 1 Sports Dentistry Association of Chile (AODCh), Santiago, Chile; 2 Faculty of Medicine, Universidad del Desarrollo, Santiago, Chile; 3 Faculty of Medicine, Universidad Señor de Sipan, Chiclayo, Peru; 4 Universidad Norbert Wiener, Lima, Peru

**Keywords:** Dentoalveolar trauma, athletes, prevalence, multi-sport events, tooth injuries, Latin America.

## Abstract

**Introduction:**

Severe damage to teeth or the periodontium can significantly affect oral functions of athletes during sport games reducing their performance and participation. We aimed to determine the prevalence of dentoalveolar trauma in high-performance athlete participating in multi-sport events in Latin American countries.

**Methods:**

A comprehensive search was performed between December 15–22, 2024 in 12 databases including following the PRISMA guidelines and Robvis for bias assessment. The inclusion criteria encompassed original articles, correspondences, scientific letters, prevalence studies, and studies on dentoalveolar trauma in athletes during multi-sport events in Latin America.

**Results:**

The initial literature search yielded a total of 798 articles. The remaining articles underwent full-text review, leading to the inclusion of one article for analysis. The selected study was authored by Enrique Amy, a researcher from the University of Puerto Rico School of Medi-cine. Published in the Dental Traumatology Journal in 2005, the study aimed to investigate the incidence of dental and orofacial trauma among athletes participating in the Central American and Caribbean Games across six different editions. The main reasons for seeking dental care among the 279 athletes included mouthguard fabrication, resolution of acute dental and orofacial conditions, restorations, and dental evaluations. Detailed information on the specific injuries and sports associated with the athletes is provided, including 18 cases of treated acute dental conditions, which primarily occurred during sport-related activities and accounted for 6% of all dental consultations during the games.

**Conclusions:**

This systematic review provides valuable insights into the prevalence of dentoalveolar trauma in high-performance athletes participating in multi-sport events in Latin America. The findings highlight the importance of preventive measures, such as mouthguard use, to mitigate the risk of dental and orofacial injuries in this population.

## Introduction

The recent increase in the standard of living and leisure time has resulted in a rise in sports participation among the population. ^1^ While sports involvement is associated with numerous health benefits, there is also a risk of injuries. ^2^ Traumatic injuries are a significant health problem in populations worldwide. With the growing number of new athletes and the popularity of sports, the incidence of sports-related injuries continues to rise. ^3^ Although the incidence of such injuries is widely reported, there are differences among studies in terms of case definitions, ^4^ and the prevalence of dental injuries also varies depending on the type of sport practiced. ^5^ Prevalence rates of dental traumas previously reported ^6^ range from 8% to 45%, and men are two to three times more likely to suffer dental traumas in permanent teeth (12% to 33%) compared to women (4% to 11%). ^7^

Injuries arising from sports activities have the potential to impact both soft and hard tissues, their severity contingent on the anatomical site and force application direction, as documented in a prior investigation. ^3^ Various contributing factors, including falls, physical altercations, sports-related incidents, vehicular accidents, and epileptic episodes, can precipitate sports-related injuries. ^6^ Given the often-accidental nature of these injuries, prompt emergency dental intervention becomes imperative. Among adults, crown fractures affecting the permanent maxillary anterior teeth emerge as the most prevalent form of dental trauma, encompassing nearly 79% of cases. ^6^ Such fractures may entail damage limited to enamel, extend to both enamel and dentin, or involve enamel, dentin, and pulp. ^7^

Severe damage to teeth and/or the periodontium can significantly affect oral functions, including speech and chewing, in addition to having a negative impact on psychosocial development due to aesthetic issues. ^8^ Baiju et al. further emphasized that oral health problems can have a variety of negative impacts on overall well-being, reduce the quality of life, and hinder athletic performance. ^9^ Traumas to the oral and maxillofacial region can also result in problems such as reduced participation in sports games, ^1^ making it crucial to consider them during sports competitions. ^10^

Diverse classification systems have been employed to characterize traumatic injuries, leading to inconsistent outcomes in some prevalence studies. ^6^ Retrospective questionnaires are commonly used in studies examining oral trauma, ^5^ and accurately estimating the prevalence and patterns of dental injuries, as well as identifying associated factors, can inform public health policies and strategies in this area. ^11^

The occurrence of dental trauma in elite athletes highlights the importance of conducting epidemiological research within this population to better understand the nature and extent of such injuries. ^12^ Examining the prevalence of dental trauma in specific geographic regions is pivotal for recognizing the occurrence of these injuries in distinct populations. ^11^ Despite extensive research on traumatic dental injuries in various contexts, including sports, no known study has specifically investigated the prevalence of dental traumas among athletes participating in the Pan American Games. ^6^

In this study, we conducted a systematic review to determine the prevalence of dentoalveolar trauma in high-performance athletes. Another objective of the review was to describe the frequency of injuries according to the country of origin of athletes participating in multi-sport events held in Latin American countries.

## Methods

### Study design and database

**Figure 1.**
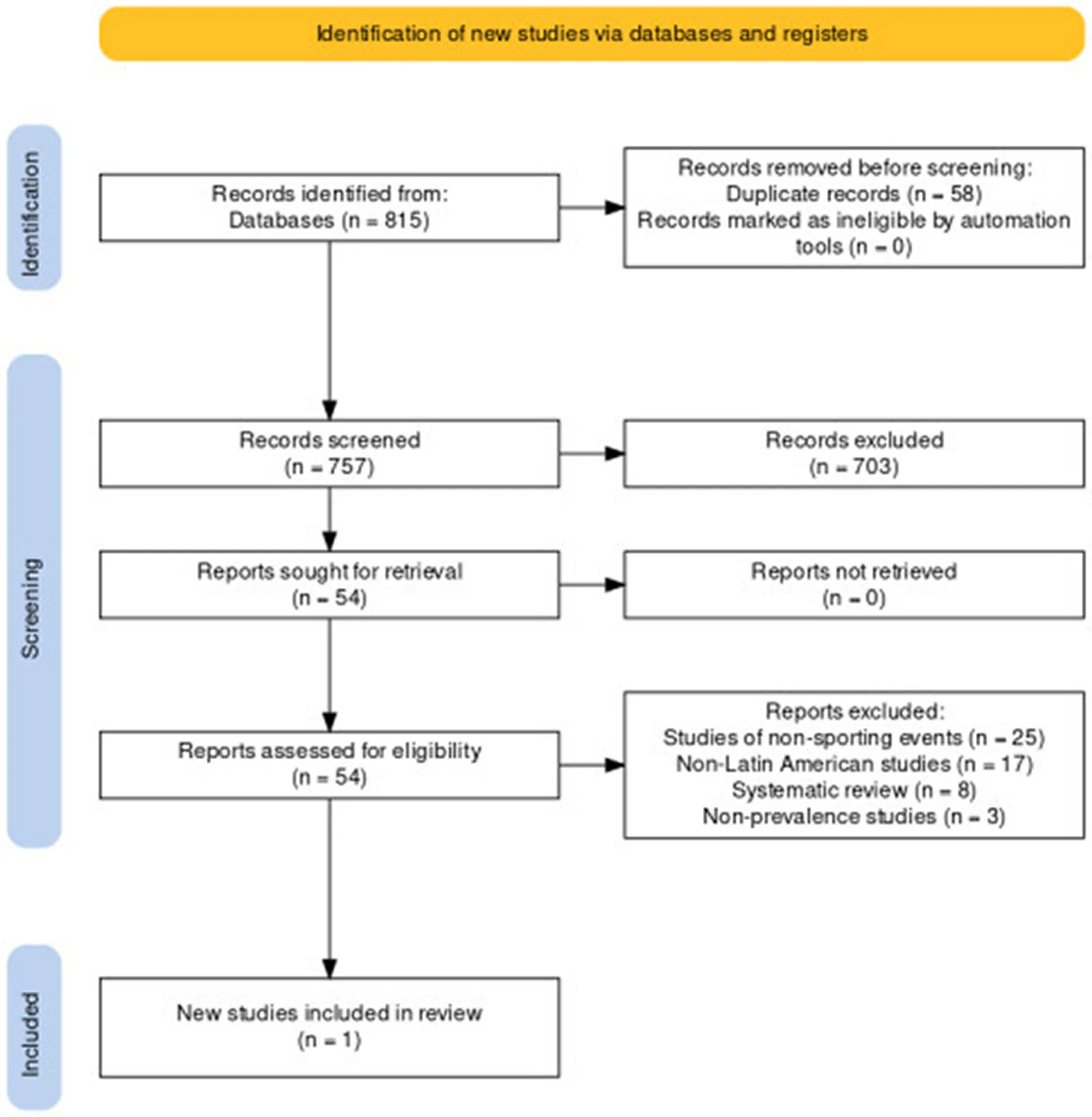
PRISMA 2020 Flowchart for the selection of studies.

We designed a systematic review following the Preferred Reporting Items for Systematic Reviews and Meta-Analysis (PRISMA) 2020 recommendations and pre-registered it in PROSPERO (CRD 451763, on August 21, 2023). The research question was formulated using the PIO acronym. ^13^ The population was high-performance athletes, the intervention was dentoalveolar trauma, and the outcomes were the prevalence of dentoalveolar trauma. The review was conducted in the following databases: Pubmed (MEDLINE), Scielo, Scopus, Wiley, ScienceDirect, EBSCO, Cochrane Library, Web of Science, and Lilacs. Additionally, we used Sportxiv, Dimensions, and Google Scholar. The identification of studies and their flow can be observed in Figure 1.

### Search strategy and inclusion criteria

For the search, we used the following Boolean descriptors and keywords (MESH terms): ((dental trauma OR oral trauma OR tooth injury OR tooth fractures OR orofacial injuries) AND (athletes OR elite athletes OR professional athletes OR high-performance) AND (Latin America OR Pan American AND (multi-sport event)). The manual search was conducted between December 15–22, 2022, and in two languages (English and Spanish).

The inclusion criteria were the following: (i) original articles, correspondence articles, and scientific letters, (ii) prevalence studies, (iii) dentoalveolar trauma studies in athletes during multi-sport events in Latin America between 1980 and 2022. We did not apply a time filter to limit the years of publication to a specific period. Moreover, in regard to exclusion criteria, we did not include the following: (i) studies from other continents, (ii) incidence or exploratory studies, (iii) dentoalveolar traumas in non-multi-sport events, and (iv) systematic reviews, meta-analyses, reflection articles, editorials, clinical cases, history articles, and method papers.

### Data extraction and quality assessment

Two authors (F.A. and J.P.M.) independently evaluated the abstracts of each article and excluded those that did not meet the inclusion criteria. Subsequently, the full text of each selected article was reviewed according to the predetermined protocol, and any disagreements between the authors were resolved through consensus at each stage of the review. Cohen’s Kappa correlation test was used to assess the overall agreement between the reviewers, revealing a good level of agreement in the search process (k = 0.811).

Data Extraction Process involved retrieving relevant information from the included studies using the CASPe (Critical Appraisal Skills Program) template of a database in MS-Excel 2013 (Microsoft Corp., Redmond, WA, USA). ^14^ The possible bias of identified documents was evaluated using the Cochrane risk of bias tool (Robvis 2.0). The studies that were considered to have a high risk of bias were those that did not include at least one outcome in their analysis.

### Data analysis

We used the IBM SPSS version 23.0 (Armonk, NY, USA) for the data analysis. A descriptive analysis of the included studies was performed to estimate their respective frequencies. Due to heterogeneity among the identified documents, it was not possible to conduct a meta-analysis, and instead, a narrative analysis of the research, focused on the primary findings of each included study, was presented.

## Results

### Search results

Initially, an extensive literature search was conducted using several databases, including PubMed (n = 4), Scopus (n = 43), Wiley (n = 67), EBSCO (n = 11), Web of Science (n = 5), LILACS (n = 7), ScienceDirect (n = 20), Dimensions (n = 498), and Google Scholar (n = 160), resulting in a total of 798 articles. After eliminating 58 duplicates and articles in languages other than English or Spanish, the remaining articles were reviewed based on their titles and abstracts, excluding those irrelevant to the research question. Subsequently, the full texts of the remaining articles were carefully reviewed, leading to the exclusion of 53 studies and leaving only one article for analysis.

### Characteristics of the included study

The selected article for this study was written by Enrique Amy, ^15^ a member of the Department of Physical Medicine, Rehabilitation, and Sports Medicine at the University of Puerto Rico School of Medicine. Published in the Dental Traumatology Journal in 2005, the main objective of the study was to investigate the incidence of dental and orofacial trauma in athletes participating in the Central American and Caribbean Games in six different editions: Cuba (1982), Dominican Republic (1986), Mexico (1990), Puerto Rico (1993), Venezuela (1998), and El Salvador (2002).

**Figure 2.**
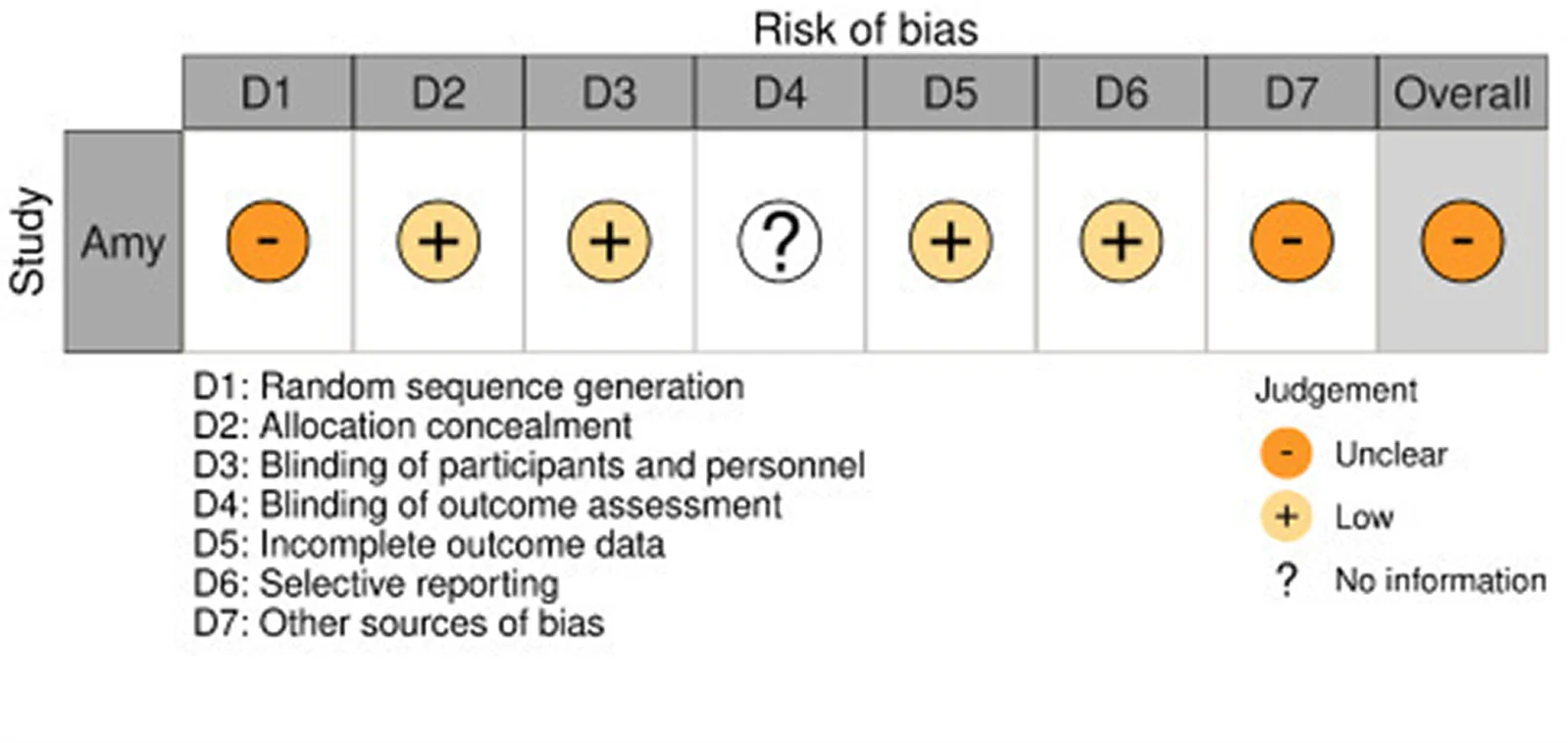
Bias assessment of the study included in this review.

Among the 2107 athletes across six delegations in various editions of these games, our study specifically examined 279 athletes seeking dental treatment and evaluation at the Medical Center of the Puerto Rico delegation. The selection of athletes was randomized. Registration details for each athlete encompassed personal information like name, age, gender, sports type, medical and dental history, along with the reason for their dental consultation. Finally, Amy’s study ^15^ presented an unclear bias, as described in Figure 2.

### Main findings

The main reasons for seeking dental care were the fabrication of mouthguards, resolution of acute dental and orofacial conditions, the need for restoration, and dental evaluations. Out of the total 279 athletes who received dental care, 133 (47.7%) were assessed for mouthguard fabrication, 18 (6.4%) for acute condition resolution, 52 (18.6%) for restorations, and 76 (27.2%) for dental evaluations. A detailed breakdown of the consultation reasons for each edition of the multi-sport event is provided in Table 1.

**Table 1.** Number of interventions in the different game events. Data in N (%).

Country (year)	Patients	Mouthguard	Acute condition	Rehabilitation	Evaluation
Cuba (1982)	48 (17.2)	0 (0)	5 (27.8)	30 (57.7)	13 (17.1)
Dominican Republic (1986)	21 (7.5)	0 (0)	0 (0)	5 (9.6)	16 (21.0)
Mexico (1990)	89 (31.9)	54 (40.6)	5 (27.8)	6 (11.5)	24 (31.6)
Puerto Rico (1993)	44 (15.8)	33 (24.8)	3 (16.7)	3 (5.8)	5 (6.6)
Venezuela (1998)	52 (18.6)	34 (25.6)	5 (27.8)	6 (11.5)	7 (9.2)
El Salvador (2002)	25 (8.9)	12 (9.0)	0 (0)	2 (3.8)	11 (14.5)
Total	279 (100)	133 (100)	18 (100)	52 (100)	76 (100)

Among the 18 instances of acute dental conditions treated, the majority (6% of all dental consultations during the games) were associated with sports activities. Dental fractures were the predominant form of dentoalveolar trauma, with specific details of injuries and the corresponding sports outlined below:

- Four instances of lip contusions occurred in karate and boxing (two each).
- Three cases of periapical injuries were reported in baseball, basketball, and athletics.
- Dental fractures were observed in two cases, one in field hockey and another in athletics.
- Two cases of lip lacerations in wrestling and basketball.
- Fractured jaws occurred in two cases, one each in basketball and baseball.
- Periapical abscesses were reported in three cases, associated with athletics, rifle shooting, and parade.
- One case of avulsion of teeth in boxing.

## Discussion

This systematic review based on Latin American studies revealed that few studies addressed the main objective. Only one study in the last four decades summarized the available published literature on the prevalence of dentoalveolar trauma in high-performance athletes. This analysis of six sports events demonstrated that mouthguard fabrication and resolution of acute dental and orofacial conditions were the primary dental care events. Additionally, the majority of cases of treated acute dental conditions were related to sports activities such as boxing, karate, and basketball. Our results reveal an area of research that requires detailed regional-wide study on dentoalveolar trauma in athletes, given its social, sporting, and economic impact.

### Strengths

First, the study begins by highlighting a comprehensive literature search, using multiple accredited databases, grey literature, and thesis collections, showcasing a thorough strategy for data acquisition. Another strength of the study, to the best of our knowledge, is that it is the first review in Latin America that addresses dental issues in athletes. The results effectively communicate the reasons for seeking dental care, the breakdown of athletes’ visits for various purposes, and a detailed description of acute dental conditions and trauma treated during sporting events.

### Main findings

The prevalence of dentoalveolar traumas varies mainly in relation to the type of sport practiced, finding very dissimilar prevalence among them, resulting in a great heterogeneity in the types of results found in relation to the variable under study. The World Dental Federation (FDI) classified sports into two categories based on the risk of dentoalveolar trauma: High risk (such as American Football, field hockey, ice hockey, lacrosse, martial arts, rugby, skates, skateboards, and mountain biking) and moderate risk (such as basketball, soccer, handball, diving, squash, gymnastics, parachuting, and water polo). ^16^ For example, in non-contact sports like volleyball, a lower prevalence of dental trauma has been reported compared to contact sports such as taekwondo and ice hockey. ^17, 18^ Similar studies have found that men are two to three times more likely to suffer some type of dental and orofacial trauma than female athletes. ^19^ On the other hand, amateur athletes tend to suffer maxillofacial injuries more frequently than professional athletes. One study demonstrated that only 10% of patients who experienced this type of injury were professional athletes. ^20^

In Amy’s study, ^15^ sports events in Cuba (1982), Dominican Republic (1986), Mexico (1990), Puerto Rico (1993), Venezuela (1998), and El Salvador (2002) were included. However, there are other sports events for which no reports on this topic have been made. As we approach the hosting of the upcoming Pan American Games in Chile 2023, there are aspects that have not been addressed in previous editions, as reviewed in the limited existing literature. The low incidence of dentoalveolar traumas found in this study may be due to the fact that the majority of “sporting hours” are spent during the practice and training of professional athletes. Unlike this study, as we analyze this variable during a short period of a multi-sport event. For example, according to a study conducted at the Lima 2019 Pan American Games, ^21^ the prevalence rate of dentoalveolar traumas was so low that it was considered one of the problems that arose during the sporting event, along with acute pain, malar bone fracture, and adjustment of orthodontic appliances, representing only 9.2% of the issues consulted during the sporting event, demonstrating that the majority of oral problems faced by athletes already existed before the start of the event.

The prevalence of dental trauma has been shown to be related to predictive factors such as the intensity and speed of the sport, the level of contact among participants, and the use and type of sports protective equipment, including mouthguards. ^22^ Several prevalence studies on dental trauma worldwide, such as the one conducted by Andrade ^6^ at the Pan American Games in Rio de Janeiro 2017, have demonstrated variation in results due to different methodologies used. Most of these studies rely on retrospective self-reporting approaches, which do not reflect the incidence at the time of the multi-sport event, making it difficult to quantitatively and qualitatively determine the variable being analyzed in this systematic review. Moreover, self-reports do not accurately qualify these sports-related injuries, introducing bias because of the absence of a competent clinician to diagnose them. Therefore, the importance of generating epidemiological research protocols regarding the variable in question during multi-sport events in Latin America becomes crucial for creating public policies aimed at prevention and treatment of these injuries related to professional athletes. Among these public policies, the creation of an assessment and action protocol aimed at the multidisciplinary management of dentoalveolar trauma is essential.

It is important to consider these differences in the prevalence and risk of dentoalveolar traumas according to the sport and level of competition. These findings can be useful in informing orodental prevention and protection strategies in athletes in order to reduce the incidence of injuries and promote oral health in this population group. Moreover, further research is required to better understand risk factors and develop sport-specific interventions.

### Limitations

In this systematic review, we have identified some limitations related to epidemiological research and management protocols regarding dentoalveolar traumas in Latin American multi-sport events. Firstly, we found limitations in the collection of specific epidemiological data and management protocols for these traumas. For instance, while dental fractures were reported as the most common type of dentoalveolar trauma, it was not specified whether these were enamel, enamel-dentin, or enamel-dentin-pulp fractures. This lack of detail hinders a precise understanding of the magnitude and severity of the traumas. Secondly, although we used meta-search engines and preprints to expand the number of evaluated studies, ^23^ we might have not included all relevant gray literature. This could mean that part of the available evidence was not taken into account in this review, which could have improved the results. Thirdly, we observed that sports events have not implemented a surveillance system that allows standardized registration and reporting of sports injuries, including dentoalveolar traumas. A Surveillance System for Injuries and Diseases (SVLE) has proven successful in recent Pan American sports events ^24^ and could be a valuable tool for registering and tracking dentoalveolar traumas in future events.

These limitations highlight the need for improved data collection and management protocols related to dentoalveolar traumas in multi-sport events. Furthermore, greater awareness is needed regarding the importance of implementing adequate surveillance systems to obtain accurate and comparable data between events. These improvements would allow a better understanding of the incidence, severity, and risk factors associated with dentoalveolar traumas, which in turn would facilitate the implementation of more effective preventive and management strategies for the benefit of athletes.

## Conclusion

the available evidence to date does not provide a definitive conclusion regarding dentoalveolar trauma in high-performance athletes in Latin American multi-sport events. The only study included in our analysis showed that most oral problems during the competition were not traumas but rather pre-existing diseases or issues. This highlights the importance of the role of sports dentists in competitions and sports teams. However, the low number of studies focused on this topic reveals a research gap and the need to address the field of sports dentistry in the Latin American region.

## Data Availability

No data are associated with this article. **Figshare:** PRISMA 2020 Check list - Frequency of dentoalveolar trauma in high-performance athletes: a systematic review in multi-sport events in Latin America, https://doi.org/10.6084/m9.figshare.31875943. ^25^ Data are available under the terms of the Creative Commons Attribution 4.0 International license (CC-BY 4.0).
