# Supplementary material for: Dental consultations and orofacial injuries in high-performance athletes participating in Latin American multi-sport events: a systematic review
Source: F1000Res. 2026 Aug 4;15:554. Originally published 2026 Apr 18. [Version 2] doi: 10.12688/f1000research.169394.2 (PMC13560792; doi:10.12688/f1000research.169394.2)
Supplement: Peer review history

# Reviewer report: Reviewer response for version 2

**DOI:** 10.5256/f1000research.204809.r509245

**Published:** 2026-08-27

**Authors:** Rodrigo Ernesto Salazar Gamarra

**Affiliations:** 1 Universidad Científica del Sur, Lima, Peru

This article examined the frequency and characteristics of dental consultations and orofacial injuries aiming high-performance athletes participating in multi-sport event is in Latin America.

 Despite the extensive search, only one study was eligible, covering 2107 athletes from six editions of the Central American and Caribbean Games of whom 279 received dental care.

 The principal finding was that the most common reasons for consultation was pre-existing rather than caused by the sporting event itself.

 Appropriate attention was given by the authors to the limitations of the study.

Is the work clearly and accurately presented and does it cite the current literature?

Yes

If applicable, is the statistical analysis and its interpretation appropriate?

Yes

Are all the source data underlying the results available to ensure full reproducibility?

Yes

Is the study design appropriate and is the work technically sound?

Yes

Are the conclusions drawn adequately supported by the results?

Yes

Are sufficient details of methods and analysis provided to allow replication by others?

Yes

Reviewer Expertise:

I have expertise in oral and maxillofacial rehabilitation, clinical research and multidisciplinary management of complex oral conditions, providing an appropriate background to asses it.

I confirm that I have read this submission and believe that I have an appropriate level of expertise to confirm that it is of an acceptable scientific standard.

# Reviewer report: Reviewer response for version 2

**DOI:** 10.5256/f1000research.204809.r507793

**Published:** 2026-08-06

**Authors:** Rodrigo Okubo

**Affiliations:** 1 Santa Catarina State University, Florianopolis, Brazil

Thank you for the opportunity to review the revised version of this article. The manuscript has improved in its acknowledgement that the available evidence is insufficient to estimate the prevalence of traumatic dental and orofacial injuries. However, several central methodological and internal-consistency issues remain unresolved.

 1. The eligibility criteria state that prevalence studies were included, whereas incidence and exploratory studies were excluded. Nevertheless, the sole included study is described in the manuscript as an investigation of the incidence of dental and orofacial trauma and appears to be based primarily on dental-service utilization. This discrepancy must be resolved by clarifying the eligible study designs and applying the criteria consistently.

 More importantly, Andrade et al. (2010), concerning the prevalence of dental trauma among Pan American Games athletes, and Opazo-García et al. (2021), concerning oral health problems at the Lima 2019 Pan American Games, are discussed in the manuscript but were not included in the review. Based on the stated population, setting, and outcomes, these studies appear potentially eligible. The authors should provide explicit study-level reasons for their exclusion. If they satisfy the eligibility criteria, the search, selection process, PRISMA diagram, data extraction, results, and conclusions must be revised accordingly. Until this issue is resolved, the conclusion that only one eligible study exists cannot be verified.

 2. The manuscript refers to the Cochrane risk-of-bias tool and “Robvis 2.0” in the assessment of a descriptive observational study. RoB 2 is intended for randomized trials and is not appropriate for the included study design. The authors should use and accurately describe an appraisal instrument suitable for cross-sectional or descriptive observational studies. If AXIS is retained, the rationale for the numerical thresholds and the interpretation of the total score should also be justified.

 3. The manuscript reports 18 acute dental and orofacial conditions, but the itemized injury categories sum to 17 occurrences. This discrepancy should be reconciled. The statement that dental fractures were the predominant form of trauma also appears inconsistent with the frequencies presented. In addition, periapical abscesses and other pre-existing dental conditions should not automatically be classified as sports-related traumatic injuries. The denominator and classification used for the reported 6% should be stated precisely.

 4. The secondary objective was to describe injuries according to the athletes’ country of origin. However, results stratified by country of origin are not presented. This objective should either be addressed with the available data or removed.

 5. The manuscript frequently uses “incidence” despite the absence of an appropriate population-at-risk and person-time denominator. The authors should distinguish incidence, prevalence, frequency of consultations, and proportion of dental-service users throughout the article. The reference to the Chile 2023 Pan American Games as an upcoming event must also be updated.

 The topic is relevant, and a rigorous demonstration of the scarcity of regional evidence could make a useful contribution. However, the unresolved eligibility and study-selection issues directly affect the central result and conclusion of the review. A substantially revised version could be reconsidered after the review corpus, methods, and numerical inconsistencies have been fully reconciled.

Is the work clearly and accurately presented and does it cite the current literature?

Partly

If applicable, is the statistical analysis and its interpretation appropriate?

Partly

Are all the source data underlying the results available to ensure full reproducibility?

Partly

Is the study design appropriate and is the work technically sound?

Partly

Are the conclusions drawn adequately supported by the results?

Partly

Are sufficient details of methods and analysis provided to allow replication by others?

Partly

Reviewer Expertise:

Sports injury epidemiology; sports physiotherapy and rehabilitation; musculoskeletal health; clinical research methodology; systematic reviews and evidence synthesis. My assessment focuses mainly on the methodological quality of the review, reporting transparency, interpretation of sports injury data, and whether the conclusions are supported by the results.

I confirm that I have read this submission and believe that I have an appropriate level of expertise to confirm that it is of an acceptable scientific standard, however I have significant reservations, as outlined above.

# Reviewer report: Reviewer response for version 1

**DOI:** 10.5256/f1000research.186734.r488263

**Published:** 2026-05-28

**Authors:** Endang Sjamsudin

**Affiliations:** 1 Universitas Padjadjaran, Bandung, Indonesia

1. Clarity and accuracy of presentation: Partial

 The abstract states the search was conducted in December 2024, while the methods list December 2022. Furthermore, the article states that studies from 1980–2022 were included, but also states there was no time limit. The cited literature is quite relevant, but not yet fully “up-to-date.” Some key references are outdated.

 2. Research design and technical quality: Partial

 Because only one study was included, this article is more accurately described as a narrative synthesis of limited evidence than a systematic review capable of estimating prevalence.

 The included study is not truly a population prevalence study of athletes, but rather data on dental consultations among athletes who visited a healthcare provider. The denominator is not all athletes at risk, but rather athletes seeking dental care. Therefore, the term “prevalence” requires great care.

 3. Detailed methods and analysis for replication: Partial

 The methods are not detailed enough to be replicated. The authors cite 12 databases/sources but do not provide complete search strings for each. The Boolean strategy is also ambiguous, particularly the combination "Latin America OR Pan American AND multi-sport event." Furthermore, the PRISMA numbers are inconsistent: the flowchart shows 815 records, while the results list 798 articles. This needs to be corrected.

 4. Statistical analysis and interpretation: Partial

 The use of SPSS is not very meaningful in this context. The analysis should be described as descriptive narrative, not as if there were a complex statistical analysis. The interpretation also needs improvement. Data on 18 cases of acute conditions from 279 dental consultations should not be directly interpreted as the prevalence of dentoalveolar trauma in all athletes.

 5. Availability of source data: Partial

 The article states no source data for this article but provides a PRISMA checklist on Figshare.

 6. Are the conclusions supported by the results? Partial

 Some statements in the abstract are too strong, for example, stating that this review provides insight into prevalence. With only one study based on dental consultations, the article cannot yet draw conclusions about population prevalence.

 Points to improve for scientific validity of the article

 Authors need to clarify the primary outcome: whether the assessment is trauma prevalence, dental consultation frequency, or trauma frequency among dental service users. Authors also need to correct inconsistencies in search dates, number of articles, and inclusion period.

 Recommended review decision:

 Major revision / approved with reservations. The article is worthy of consideration but requires substantial methodological and interpretative revisions before it can be considered scientifically sound.

Is the work clearly and accurately presented and does it cite the current literature?

Partly

If applicable, is the statistical analysis and its interpretation appropriate?

Partly

Are all the source data underlying the results available to ensure full reproducibility?

Partly

Is the study design appropriate and is the work technically sound?

Partly

Are the conclusions drawn adequately supported by the results?

Partly

Are sufficient details of methods and analysis provided to allow replication by others?

Partly

Reviewer Expertise:

oromaxillofacial trauma and temporomandibular joint disorders

I confirm that I have read this submission and believe that I have an appropriate level of expertise to confirm that it is of an acceptable scientific standard, however I have significant reservations, as outlined above.

# Response

**Published:** 2026-06-24

**Authors:** Jeel Moya-Salazar

**Affiliations:** GRINA, Faculty of Medicine, Universidad Senor de Sipan, Chiclayo, Lambayeque, Peru

**REPONSE TO REVIEWER**

 1. Clarity and accuracy of presentation: Partial

 The abstract states the search was conducted in December 2024, while the methods list December 2022. Furthermore, the article states that studies from 1980–2022 were included, but also states there was no time limit. The cited literature is quite relevant, but not yet fully “up-to-date.” Some key references are outdated.

 RESPONSE: The text has been corrected for spelling and grammar errors, following the cited comments. Where possible, and given the lack of current evidence on the topic in the region, the references appear to be outdated; therefore, they have been updated.

 2. Research design and technical quality: Partial

 Because only one study was included, this article is more accurately described as a narrative synthesis of limited evidence than a systematic review capable of estimating prevalence.

 The included study is not truly a population prevalence study of athletes, but rather data on dental consultations among athletes who visited a healthcare provider. The denominator is not all athletes at risk, but rather athletes seeking dental care. Therefore, the term “prevalence” requires great care.

 RESPONSE: We agree with the comments, and the study's objectives, title, results, interpretation, and conclusion have been reorganized. This comment aligns with that of the other reviewer and is an important point to consider, as a single study and the available evidence are insufficient to estimate prevalence. This has also been acknowledged in the limitations.

 3. Detailed methods and analysis for replication: Partial

 The methods are not detailed enough to be replicated. The authors cite 12 databases/sources but do not provide complete search strings for each. The Boolean strategy is also ambiguous, particularly the combination "Latin America OR Pan American AND multi-sport event." Furthermore, the PRISMA numbers are inconsistent: the flowchart shows 815 records, while the results list 798 articles. This needs to be corrected.

 RESPONSE: The wording has been reviewed and the search terms used have been updated in both Spanish and English. Inconsistencies in the text have also been addressed.

 4. Statistical analysis and interpretation: Partial

 The use of SPSS is not very meaningful in this context. The analysis should be described as descriptive narrative, not as if there were a complex statistical analysis. The interpretation also needs improvement. Data on 18 cases of acute conditions from 279 dental consultations should not be directly interpreted as the prevalence of dentoalveolar trauma in all athletes.

 RESPONSE: This section has been corrected, and descriptive-narrative analysis has been adopted as the primary approach. Additionally, the study did not estimate prevalence because only one study was found; therefore, the scope has changed. Please review the adjustments.

 5. Availability of source data: Partial

 The article states no source data for this article but provides a PRISMA checklist on Figshare.

 RESPONSE: The data sources are indicated in the article and the PRISMA checklist for this study is already described in the “Reporting Guidelines” section and can be consulted at: https://doi.org/10.6084/m9.figshare.31875943

 6. Are the conclusions supported by the results? Partial

 Some statements in the abstract are too strong, for example, stating that this review provides insight into prevalence. With only one study based on dental consultations, the article cannot yet draw conclusions about population prevalence.

 RESPONSE: We agree with the reviewer on this point, and the entire document has been adjusted to be more specific regarding the prevalence estimate/dental visits.

 Points to improve for scientific validity of the article

 Authors need to clarify the primary outcome: whether the assessment is trauma prevalence, dental consultation frequency, or trauma frequency among dental service users. Authors also need to correct inconsistencies in search dates, number of articles, and inclusion period.

 RESPONSE: The main outcome of the study is the frequency of trauma among users of dental services. Furthermore, errors in the text and consistency of the stated objectives have been corrected. We appreciate your review and believe it has improved the quality of the revision.

# Reviewer report: Reviewer response for version 1

**DOI:** 10.5256/f1000research.186734.r477053

**Published:** 2026-05-05

**Authors:** Rodrigo Okubo

**Affiliations:** 1 Santa Catarina State University, Florianopolis, Brazil

**Overall assessment**

 The article addresses a relevant and underexplored topic: the frequency of dentoalveolar trauma among high-performance athletes participating in multi-sport events in Latin America. The review is potentially useful because oral and maxillofacial injuries may affect athletes’ health, participation, performance, and the planning of preventive strategies during sports events. The manuscript also has some strengths, including the attempt to search multiple databases and grey literature sources, the use of a PRISMA flow diagram, and a clear recognition that the available evidence in this specific field is scarce.

 However, I have several methodological and reporting concerns that should be addressed before the article can be considered scientifically sound.

**Major points that must be addressed**

 1. The manuscript states that the aim was to determine the prevalence of dentoalveolar trauma in high-performance athletes participating in multi-sport events in Latin America. However, the only included study appears to report dental consultations and acute dental/orofacial conditions among athletes seeking dental care, rather than prevalence in the entire athlete population. Therefore, the authors should clarify whether the review is estimating prevalence, incidence, frequency of consultations, or frequency of trauma among dental-service users. If the available evidence does not allow prevalence to be estimated, this should be explicitly stated in the title, abstract, results, discussion, and conclusion.

 2. There are important inconsistencies in the manuscript. The abstract states that the search was conducted between December 15–22, 2024, whereas the Methods section reports December 15–22, 2022. The Methods also state that studies from 1980 to 2022 were eligible, while also saying that no time filter was applied. These inconsistencies make it difficult to understand the actual search period and eligibility window. The authors should provide a single, consistent account of the search date, eligible publication period, and whether any date restriction was applied.

 3. The review reports that 12 databases/sources were searched, but the exact search strategies for each database are not fully provided. The Boolean expression also appears incomplete or ambiguous, particularly regarding the use of “Latin America OR Pan American AND multi-sport event”. The authors should provide the complete search strings for all databases, including Spanish terms if the search was performed in English and Spanish, as well as the date of each search, filters used, and the number of records retrieved from each source. For Google Scholar, the authors should state how many pages or results were screened.

 4. The flow diagram reports 815 records identified, whereas the Results section reports 798 articles. This discrepancy should be corrected. In addition, because only one article was included, the authors should provide a supplementary table listing the studies excluded after full-text assessment and the specific reason for exclusion. This is particularly important because some cited studies appear highly relevant to the topic, such as studies on dental trauma or oral health problems during Pan American Games. The authors should explain why these studies were excluded or whether the eligibility criteria should be reconsidered.

 4. The use of the Cochrane risk-of-bias tool/Robvis appears inappropriate for the included study design. The domains shown in the figure, such as random sequence generation, allocation concealment, and blinding, are designed for randomized trials and do not fit observational prevalence or service-utilization studies. The authors should replace this assessment with an instrument suitable for prevalence or observational studies, such as the JBI checklist for prevalence studies, the AXIS tool, or another justified tool. The risk-of-bias judgments should then be reinterpreted accordingly.

 5. The manuscript reports that the included study involved 279 athletes seeking dental treatment and evaluation among 2107 athletes across six delegations. This is an important distinction: the denominator for trauma prevalence should be the population at risk, not only those seeking dental care. The authors should clearly distinguish between:

 a) all athletes participating in the events;

 b) athletes from the Puerto Rico delegation;

 c) athletes who sought dental care; and

 d) athletes with acute dental/orofacial conditions.

 The current wording risks overinterpreting dental-service data as population prevalence.

 6.The manuscript reports using SPSS and descriptive analysis. Given that only one study was included and no meta-analysis was performed, the statistical analysis is minimal. This is not necessarily a problem, but the authors should avoid implying that statistical procedures beyond descriptive summaries were conducted. The analysis should be presented transparently as a narrative synthesis with descriptive extraction of frequencies.

 7. The abstract conclusion states that the review provides valuable insights into the prevalence of dentoalveolar trauma and highlights preventive measures such as mouthguard use. However, with only one included study, and with data based on dental consultations rather than population-based prevalence, the conclusions should be more cautious. The final conclusion is more balanced than the abstract, but both should be harmonized. The authors should emphasize that the evidence is insufficient to estimate prevalence and that the main finding is the lack of robust epidemiological data in Latin American multi-sport events.

**Minor points**

- The PIO framework is not ideal as written because “dentoalveolar trauma” is not an intervention. The authors may consider using PECO/PEO or simply defining Population, Exposure/Condition, and Outcome.
- The manuscript should be carefully edited for grammar and consistency. For example, “high-performance athlete participating” should be revised to “high-performance athletes participating”.
- The term “dentoalveolar trauma” should be consistently defined, including whether soft-tissue injuries, jaw fractures, periapical abscesses, and orthodontic appliance issues are included or excluded.
- The manuscript should avoid statements such as “first review in Latin America” unless this claim is fully supported by the search and eligibility criteria.
- The reference to the Pan American Games in Rio de Janeiro should be checked, as the cited study appears to refer to the 2007 Pan American Games rather than 2017.
- The table title “Number of interventions” could be clearer, for example: “Reasons for dental consultations across different editions of the Central American and Caribbean Games”.

Is the work clearly and accurately presented and does it cite the current literature?

Partly

If applicable, is the statistical analysis and its interpretation appropriate?

Partly

Are all the source data underlying the results available to ensure full reproducibility?

Partly

Is the study design appropriate and is the work technically sound?

Partly

Are the conclusions drawn adequately supported by the results?

Partly

Are sufficient details of methods and analysis provided to allow replication by others?

Partly

Reviewer Expertise:

Sports injury epidemiology; sports physiotherapy and rehabilitation; musculoskeletal health; clinical research methodology; systematic reviews and evidence synthesis. My assessment focuses mainly on the methodological quality of the review, reporting transparency, interpretation of sports injury data, and whether the conclusions are supported by the results.

I confirm that I have read this submission and believe that I have an appropriate level of expertise to confirm that it is of an acceptable scientific standard, however I have significant reservations, as outlined above.

# Response

**Published:** 2026-06-24

**Authors:** Jeel Moya-Salazar

**Affiliations:** GRINA, Faculty of Medicine, Universidad Senor de Sipan, Chiclayo, Lambayeque, Peru

**REPONSE TO REVIEWER**

 Major points that must be addressed

 1. The manuscript states that the aim was to determine the prevalence of dentoalveolar trauma in high-performance athletes participating in multi-sport events in Latin America. However, the only included study appears to report dental consultations and acute dental/orofacial conditions among athletes seeking dental care, rather than prevalence in the entire athlete population. Therefore, the authors should clarify whether the review is estimating prevalence, incidence, frequency of consultations, or frequency of trauma among dental-service users. If the available evidence does not allow prevalence to be estimated, this should be explicitly stated in the title, abstract, results, discussion, and conclusion.

 RESPONSE: We greatly appreciate your comments and agree with this point. We have reviewed the entire text and adjusted the title, objectives, results, and conclusions accordingly.

 2. There are important inconsistencies in the manuscript. The abstract states that the search was conducted between December 15–22, 2024, whereas the Methods section reports December 15–22, 2022. The Methods also state that studies from 1980 to 2022 were eligible, while also saying that no time filter was applied. These inconsistencies make it difficult to understand the actual search period and eligibility window. The authors should provide a single, consistent account of the search date, eligible publication period, and whether any date restriction was applied.

 RESPONSE: This observation aligns with that of the other reviewer, and we fully concur regarding the writing errors in the text. The text has been revised and adjusted for greater clarity regarding the methodology used.

 3. The review reports that 12 databases/sources were searched, but the exact search strategies for each database are not fully provided. The Boolean expression also appears incomplete or ambiguous, particularly regarding the use of “Latin America OR Pan American AND multi-sport event”. The authors should provide the complete search strings for all databases, including Spanish terms if the search was performed in English and Spanish, as well as the date of each search, filters used, and the number of records retrieved from each source. For Google Scholar, the authors should state how many pages or results were screened.

 RESPONSE: We have reviewed the text and corrected it according to the reviewer's suggestions, including the search strings and dates.

 4. The flow diagram reports 815 records identified, whereas the Results section reports 798 articles. This discrepancy should be corrected. In addition, because only one article was included, the authors should provide a supplementary table listing the studies excluded after full-text assessment and the specific reason for exclusion. This is particularly important because some cited studies appear highly relevant to the topic, such as studies on dental trauma or oral health problems during Pan American Games. The authors should explain why these studies were excluded or whether the eligibility criteria should be reconsidered.

 RESPONSE: The exact number of reviewed articles has been adjusted. We disagree with the author on this point, as the PRISMA guidelines do not require structured reporting of articles not included in the results section. This would generate unnecessary citations of articles that are not being analyzed and that do not contribute to the study.

 4. The use of the Cochrane risk-of-bias tool/Robvis appears inappropriate for the included study design. The domains shown in the figure, such as random sequence generation, allocation concealment, and blinding, are designed for randomized trials and do not fit observational prevalence or service-utilization studies. The authors should replace this assessment with an instrument suitable for prevalence or observational studies, such as the JBI checklist for prevalence studies, the AXIS tool, or another justified tool. The risk-of-bias judgments should then be reinterpreted accordingly.

 RESPONSE: We considered using the AXIS tool as a complement to our previous bias analysis. This is because it does not provide an official risk of bias rating. This can be supplemented with the generic Robvis tool. We did not use JBI because the study does not report prevalence. We appreciate the suggestion and have attached the AXIS checklist as supplementary material.

 5. The manuscript reports that the included study involved 279 athletes seeking dental treatment and evaluation among 2107 athletes across six delegations. This is an important distinction: the denominator for trauma prevalence should be the population at risk, not only those seeking dental care. The authors should clearly distinguish between:

 a) all athletes participating in the events;

 b) athletes from the Puerto Rico delegation;

 c) athletes who sought dental care; and

 d) athletes with acute dental/orofacial conditions.

 The current wording risks overinterpreting dental-service data as population prevalence.

 RESPONSE: As suggested by both reviewers, prevalence is not a final outcome considered in this review. Therefore, the general population was not considered to estimate the impact of oral trauma. The results have been improved for clarity; please review.

 6.The manuscript reports using SPSS and descriptive analysis. Given that only one study was included and no meta-analysis was performed, the statistical analysis is minimal. This is not necessarily a problem, but the authors should avoid implying that statistical procedures beyond descriptive summaries were conducted. The analysis should be presented transparently as a narrative synthesis with descriptive extraction of frequencies.

 RESPONSE: We agree with the review on this point and recommend a narrative synthesis and frequency estimate of the study found, as the study cannot estimate prevalence with a single document. Please review changes

 7. The abstract conclusion states that the review provides valuable insights into the prevalence of dentoalveolar trauma and highlights preventive measures such as mouthguard use. However, with only one included study, and with data based on dental consultations rather than population-based prevalence, the conclusions should be more cautious. The final conclusion is more balanced than the abstract, but both should be harmonized. The authors should emphasize that the evidence is insufficient to estimate prevalence and that the main finding is the lack of robust epidemiological data in Latin American multi-sport events.

 RESPONSE: We have reviewed both conclusions and adjusted them to reflect the findings of the study. The recommendations provided have been followed.

 Minor points

 The PIO framework is not ideal as written because “dentoalveolar trauma” is not an intervention. The authors may consider using PECO/PEO or simply defining Population, Exposure/Condition, and Outcome.The manuscript should be carefully edited for grammar and consistency. For example, “high-performance athlete participating” should be revised to “high-performance athletes participating”.The term “dentoalveolar trauma” should be consistently defined, including whether soft-tissue injuries, jaw fractures, periapical abscesses, and orthodontic appliance issues are included or excluded. The manuscript should avoid statements such as “first review in Latin America” unless this claim is fully supported by the search and eligibility criteria.The reference to the Pan American Games in Rio de Janeiro should be checked, as the cited study appears to refer to the 2007 Pan American Games rather than 2017. The table title “Number of interventions” could be clearer, for example: “Reasons for dental consultations across different editions of the Central American and Caribbean Games”.

 RESPONSE: We have adjusted the text according to your suggestions.
